# Pediatric Palliative Care in Infants and Neonates

**DOI:** 10.3390/children5020021

**Published:** 2018-02-07

**Authors:** Brian S. Carter

**Affiliations:** 1 University of Missouri-Kansas City School of Medicine, 2411 Holmes Street, Kansas City, MO 64108, USA; bscarter@cmh.edu; Tel.: +1-816-701-5268; Fax: +1-816-302-9965; 2 Children’s Mercy Hospital, Kansas City, MO 64108, USA; 3 Division of Neonatology and Bioethics Center, 2401 Gillham Road, Kansas City, MO 64108, USA

**Keywords:** neonatal, palliative care, comfort care, pain

## Abstract

The application of palliative and hospice care to newborns in the neonatal intensive care unit (NICU) has been evident for over 30 years. This article addresses the history, current considerations, and anticipated future needs for palliative and hospice care in the NICU, and is based on recent literature review. Neonatologists have long managed the entirety of many newborns’ short lives, given the relatively high mortality rates associated with prematurity and birth defects, but their ability or willingness to comprehensively address of the continuum of interdisciplinary palliative, end of life, and bereavement care has varied widely. While neonatology service capacity has grown worldwide during this time, so has attention to pediatric palliative care generally, and neonatal-perinatal palliative care specifically. Improvements have occurred in family-centered care, communication, pain assessment and management, and bereavement. There remains a need to integrate palliative care with intensive care rather than await its application solely at the terminal phase of a young infant’s life—when s/he is imminently dying. Future considerations for applying neonatal palliative care include its integration into fetal diagnostic management, the developing era of genomic medicine, and expanding research into palliative care models and practices in the NICU.

## 1. Introduction

The provision of palliative or hospice care for newborn infants was first introduced in the 1980s. At the time, hospice principles were being disseminated in the US and their applicability to certain newborns and young infants was noted by Whitfield et al. [1] and by Silverman [2]. Since 1982, there has been increased growth in the field of palliative care in general, and in both pediatric and neonatal palliative care specifically. Indeed, the specialty of palliative and hospice medicine has gained recognition in North America and internationally with the attendant growth of training programs in both the clinical and academic arenas.

## 2. Materials and Methods

A brief, but pertinent, review of the past 10 years’ clinical literature was conducted to examine the breadth of neonatal palliative care as it is currently practiced. Throughout this process, additional clinical needs, potential research ideas, and future considerations were determined and are herein addressed.

## 3. Results

As the specialty of neonatology has grown around the world in the last three decades, the role for palliative care (PC) has likewise been recognized in the neonatal intensive care unit (NICU) and pediatric intensive care unit (ICU)—both common locales for neonatal and infant mortality [3,4,5]. Over the past 15 years, marked improvements have occurred around the circumstances in which newborn and young infants die in North American hospitals. There has been attention drawn to patient needs such as pain and symptom management [6,7], spiritual support [8], honoring cultural practices [5], understanding grief [9], and employing a breadth of bereavement activities and services [10,11].

Some particular features of providing PC for newborns and young infants include how pregnancy is unique among human experiences and the veritable uniqueness of each pregnancy—and child—from all others. Mothers and fathers generally enter into pregnancy with anticipation and hopefulness, having a future-oriented idea of their yet to be born infant. That a newborn may be critically ill, extremely premature, or born with significant birth anomalies that threaten his/her life and wellbeing is never truly anticipated. When discovered—at birth or even beforehand with fetal diagnostics—the pregnancy generally takes a dramatic turn as hopeful anticipation is replaced by fear, joy may be eclipsed by guilt, and the experience of pregnancy and childbirth becomes medicalized—often with obsessive thoughts surrounding each clinic visit, imaging study, or test. In addition to cure-oriented and life-extending neonatal intensive care, the provision of concurrent PC may provide supportive care for the patient and family, and may help in decision-making [12,13].

As is true in other areas of pediatric PC, neonatal PC has had a somewhat divided history. In the NICU, there have long been a number of patients who have been treated for weeks to months only to reach a plateau or stagnation in their progress toward growth and healing. They remain ventilated, perhaps dependent upon intravenous nutrition, have endured infections and maybe bowel problems, their livers are impacted by cholestasis, and they may even have parenteral nutrition-associated liver disease. Some have endured brain injury. Each day their care is challenging, and on any given day they might have an acute decompensation for which escalating support is required—often accompanied by analgesia and/or sedation to minimize cardiopulmonary instability or neurologic agitation. These infants may further decompensate or develop secondary pulmonary arterial hypertension along with their bronchopulmonary dysplasia (BPD). Any given infection may take their life. For these infants—some of whom may linger, and others may in fact be dying—neonatal PC may be an adjunctive care paradigm that is added to their continued intensive care. For some, the cure-oriented care may yield to a palliative paradigm after intensive counseling of parents, exploration of options with the interdisciplinary neonatal team, and thoughtful reflection. Psychosocial support for the family is essential and may attend to anticipatory grief; spiritual support may increase, and a decision to limit ongoing life-support may result in a mutually agreed upon redirection of care towards comfort with a reduction in vital-sign and invasive monitoring, phlebotomies, and imaging tests. In time, a compassionate withdrawal of life-supportive technology is performed, and caregivers work with families to orchestrate a meaningful time with extended family, rituals, and allow for the infant’s passing. In such cases, a period of focused palliation culminates in veritable hospice care in the NICU, typically associated with the withdrawal of life-sustaining medical treatments.

**Figure 1:**
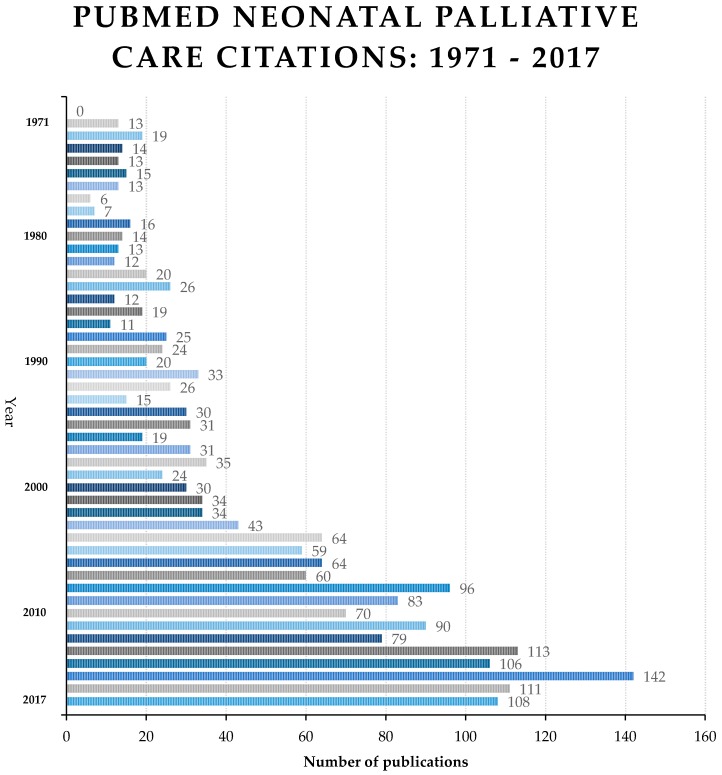
Increasing publications addressing neonatal palliative care.

As neonatal palliative care has developed, and an expanding literature is at the disposal of clinicians (Figure 1), there has been greater consideration for PC in the NICU. In recent years, especially as prenatal diagnostics have improved and life-limiting conditions are diagnosed prenatally, some newborn infants will receive concurrent palliative care (even beginning with conversations before birth) while being cared for in the NICU [14,15,16]. The care may be oriented toward confirming a prenatal diagnosis and exploring care options, or be directed at the baby’s comfort while not taking on intensive or invasive technological care. In the latter situation, time with parents, human contact, warmth, and symptom relief may be the predominant mode of support [17]. For others, such as a newborn with hypoplastic left heart syndrome, there may be intensive care provided while the neonatal, cardiology, and cardiovascular surgical specialists confer about palliative surgical options or perhaps limits or confounders (such as prematurity, additional birth defects, or the severity of anatomical size constraints that make surgery more difficult) [18]. When concurrent palliative care is made available, the added value of an interdisciplinary team, psycho-social-spiritual support, and expert pain and symptom management are acknowledged as enhancing the baby’s and family’s quality of life even while cure-oriented or disease-modifying treatments are offered. The focus is on the quality of life and relationships while living with a life-limiting condition [19,20].

A working definition for neonatal palliative care can be found from the organization *Together for Short Lives*, a children’s palliative care group in the UK:

“Palliative care for a fetus, neonate, or infant with a life-limiting condition is an active and total approach to care, from the point of diagnosis or recognition, throughout the child’s life, at the time of death and beyond. It embraces physical, emotional, social, and spiritual elements and focuses on the enhancement of quality of life for the neonatal infant and support for the family. It includes the management of distressing symptoms, the provision of short breaks, and care through death and bereavement” [21].

Note here that this involves far more than care of the imminently dying newborn. Indeed, the many domains of PC outlined by Ferrell in 2005 [22] are present and require consideration and action in the neonatal and young infant population as much as they do in adults or older children [21]. In recent years, referrals for PC from the perinatal and newborn period have often been the starting point for long-term follow-up of children with complex chronic conditions and special health care needs by PC clinicians long after the infant has left the NICU.

The types of patients for whom PC may be anticipated and offered in the newborn period are generally in one of three categories: those born at the threshold of viability or who are similarly vulnerable by virtue of prematurity, those with birth anomalies that may threaten vital functions, and those for whom intensive care has been appropriately applied but are now burdened with interventions that no longer are deemed beneficial, but come to be seen as burdensome, inappropriate, and only prolonging the infant’s dying. There are guidelines published from the National Institute of Nursing Research (NINR), National Association of Nurse Practitioners, the National Perinatal Association (NPA), the National Association of Neonatal Nurses (NANN), the National Hospice and Palliative Care Organization (NHPCO), and the Center to Advance Palliative Care (www.capc.org) that speak to these categories and potential triggers for neonatal PC [23,24,25,26,27]. For some of the commonly included conditions (below) a diagnosis may arise in the neonatal period and PC care may be initiated, whereas others may lead to a more long-term follow-up with PC as an adjunct to other ICU follow-up services:

- Genetic/chromosomal: Chromosomal aneuploidies with complex and life-limiting prognoses; severe metabolic, storage, or mitochondrial disorders; severe forms of skeletal dysplasia.
- Organ-system problems: Severe central nervous system (CNS) malformations (neural tube defects, migrational disorders); hypoxic-ischemic encephalopathy; spinal muscular atrophy type-1 and myotonic dystrophies; epidermolysis bullosa; Potter’s syndrome, fetal oligohydramnios sequence, fetal-neonatal chronic renal failure; short-gut syndrome with parenteral nutrition dependence; multi-visceral organ transplant under consideration (e.g., liver, bowel, pancreas); biliary atresia; total aganglionosis of the bowel; severe feeding impairment with feeding tube dependence that may be permanent; complex congenital heart disease, especially if functionally univentricular; extracorporeal membrane oxygenation (ECMO) patients; severe pulmonary arterial hypertension; consideration for heart transplant; congenital diaphragmatic hernia; severe pulmonary hypoplasia; congenital central hypoventilation syndrome; asphyxiating thoracic dystrophies; multi-organ system failure.
- Infection and immune disorders: Perinatal human immunodeficiency virus infection and acquired immune deficiency syndrome (HIV/AIDS); severe combined immune deficiency (SCID); severe perinatal herpes simplex virus (HSV), cytomegalovirus (CMV), toxoplasmosis or Zika virus with meningoencephalitis or severe encephalopathy.
- Complications of prematurity: Periviable gestation; severe intraventricular hemorrhage (IVH, grade IV) or periventricular leukomalacia (PVL); refractory respiratory failure; ventilator-dependent BPD; severe necrotizing enterocolitis (NEC) with resultant short gut; liver failure.

## 4. Discussion

Today, newborns and young infants may receive PC that is initiated prenatally, introduced at delivery, or acquired in a consultative manner in the NICU. The acceptance of this paradigm of care, and the capacity for it to be rendered as needed, however, varies across units of care and internationally. A number of investigators have spoken to barriers to neonatal-perinatal PC such as suboptimal interdisciplinary collaboration in hospitals, unacceptance of PC broadly in society and specifically as it applies to children, poor clinician communication skills, and prognostic uncertainties among others [28,29,30,31]. Clinicians today must strive to advance PC education, employ successful models of neonatal-perinatal PC, and incorporate PC competencies into those considered fundamental in neonatal-perinatal professional education [15,32,33,34,35,36].

Looking ahead, I will outline what I see as three major considerations for the field of PC as it pertains to pregnancy, fetal, and neonatal patients in the next decade. First, given the improved diagnostic capabilities present with maternal-fetal imaging, and the expansion of fetal diagnostic and intervention centers, the integration of PC with prenatal counseling will likely increase. For the pediatric PC specialist familiar with neonatal-perinatal care, this will not necessarily pose concerns. However, for those unfamiliar with neonatal-perinatal care (e.g., a pediatric oncologist who has been trained in PC, or a family physician now boarded in Hospice and Palliative Medicine) there may be a learning curve to ramp up capabilities to address the often unique aspects of pregnancy and childbirth complicated by life-limiting conditions [37,38,39].

In such instances where a palliative medicine consultant has little experience with newborns, it will be beneficial for neonatologists, pediatricians, and hospitalist pediatricians to be identified as local “champions” for neonatal PC within a given NICU, academic faculty, or practice group. The NICU champion will likely work with unit-specific interdisciplinary staff and the local PC-trained physician or nurse practitioner to effect well-coordinated and effective PC in the NICU. The coordination of transitional care from the NICU to home-based hospice care, an inpatient hospice-house or facility, or even outpatient management anticipated to be beneficial for months to years that is shared with a primary care pediatrician, specialty clinics, and interim contact with the PC team first met in the NICU, is of added value to both NICU staff and patient families.

Related to this first consideration is the expansion of fetal and neonatal diagnostic evaluations with genomic medicine to better understand or explain multiple anomalies, metabolic disorders, or complex central nervous system dysfunction [40,41,42,43]. I foresee that pediatric (and neonatal) clinicians in general, and perhaps PC clinicians specifically, will need to develop a facility with both new diagnostic genetic tests and the cautious compilation of new diagnostic groups with yet to be determined prognoses. While some such cases may be treatable with present regimens, others may require yet to be developed, personalized pharmacogenomics-derived treatments. In the prenatal setting, genetic/genomic diagnoses that can be made from chorionic villous sampling, amniocentesis, fetal blood sampling, or even non-invasive maternal (blood) testing will likely accompany phenotypic diagnoses seen on imaging. These developments will impose the need for expanded perinatal counseling and open doors for PC clinicians to join neonatologists, geneticists, and maternal-fetal medicine specialists in contributing to perinatal and neonatal decision-making. Already, we see the possibility of expanded genome-wide newborn screening being proposed in the care of all newborns, and even if this is constrained to symptom-driven circumstances, the need to address prognostic uncertainty will call upon the superlative communication skills of trained and informed neonatologists and PC clinicians [37,38,39,44].

As a second consideration, PC material is increasingly being incorporated into the training and education of neonatologists. This will likely result in an enhanced momentum for PC services to be better integrated into the newborn’s care concurrently with cure-oriented or life-prolonging critical care. Progress has been made in the establishment of PC teams in children’s hospitals in North America. However, due to their variable staffing, composition, resource priority, and the limited number of fellowship-trained PC specialists, the ability for these teams to concurrently provide PC throughout a large children’s hospital and in the NICU may be limited. This too provides an opportunity for neonatologists to become local champions for PC in their NICU—and in so doing building the capacity for PC as they partner with PC team members—until capacity is increased with more fellowship-trained pediatric or neonatal PC specialists. Given the longstanding interdisciplinary nature of the care team in the NICU, unit-specific neonatal nurse practitioners who may be PC champions, respiratory therapists, perinatal social workers, nurses, and others can work with chaplains, psychologists, and child-life specialists to create NICU-PC teams that address unit needs with input and assistance from pediatric PC specialists elsewhere in the hospital. Together these complimentary and concurrent teams can see to patients, family, and staff needs [19,37,38,39,44,45].

A third consideration that I would highlight in the next 10 years of PC provided for newborns is the need to conduct research, qualitative and empirical, that can advance the derivation of best practice models for neonatal PC [15,32,33,34,35,36]. Such research will advance the evidence base for the field, and likely result in increasing acceptance and utilization of PC in the neonatal-perinatal world. Investigations might address symptom assessment and management skills; studying commonly used, but poorly evaluated medications; looking further into the barriers and facilitators of PC in the NICU across staff/disciplines [28,29,30,31,39,44,45]; and evaluating effective communication strategies and decision-support for families and staff [46,47]. The utilization of large neonatal population databases to make inquiries and construct studies to answer common problems would seem to hold great potential [48]. What are the best ways to manage common symptoms? How should ventilator withdrawal be accomplished [49]? What do parents think about the limitation or withdrawal of medically assisted nutrition and hydration [50]? How can neonatologists, maternal-fetal medicine physicians, geneticists, and others best inform a pregnant couple of unexpected findings on fetal imaging or address prognosis in the NICU [51]? Would a complimentary presence of an ethics consultant add value anywhere along the path from diagnosis to treatment decisions or life-sustaining medical treatment withdrawal [52,53]?

## 5. Conclusions

The history of neonatology includes periods in which clinicians did not believe infants felt pain—now, pain assessment and management are part of standard care. Likewise, the provision of assisted ventilation, surgical interventions, and other technology at the neonatologist’s disposal have all increased neonatal survival, and improved patients’ quality of life through the NICU’s practicing developmentally supportive care. However, when technology becomes more burdensome than beneficial, when a newborn’s relational potential seems lost due to severe neurologic injury, or when being too premature and too small—or fraught with too many anomalies—confronts families and clinicians with the limits of medicine, PC should be at the ready (or already have been incorporated into a concurrent care model) to assist all stakeholders. I believe that the foundation for the integration of PC into the NICU is set, the need is present, and the progress made over the past 15 years speaks to a bright future in the practice of humane, ethical, and family-centered care for our world’s tiniest and most vulnerable patients. We can meet the challenges outlined here and others with continued efforts, well-trained young leaders, forthcoming research, appropriate advocacy, and local hospital and university support.
